# Keeley in the Pulpit

**Published:** 1892-06

**Authors:** 


### KEELEY IN THE PULPIT.

The papers state that Dr. Talmage's pulpit in Brooklyn was occupied on Sunday night, May 15th, by the notorious Keeley, of Dwight, Ill. During the evening Keeley and his press agent were entertained by the great divine at the latter's home. The "services" were opened with prayer by the Rev. I. K. Funk, who invoked heaven's choicest benedictions upon the precious head of Keeley. Talmage introduced him to his audience as the one who, by the grace of God, would rescue a fallen world from the sin and degradation of the whiskey and opium habits. We may easily imagine how he utilized this grand opportunity to inoculate his pious hearers with the supposed virtues of his so-called cure, and with the wisdom and genius and greatness of its discoverer. From this sacred eminence he declared that but three living persons knew the constituents of his cure, and that they would never divulge the secret. Assuming his treatment to possess any value, the careful guarding of his secret is the height of selfishness and avarice. Compared with this mean and base and sordid spirit one has only to examine the weekly medical press to find the thousand instances in which humble but honest and conscientious physicians cheerfully and gladly, and sometimes too quickly, announce to the world some "discovery" of theirs which may be of benefit to the sick and suffering. But this man is now become a god, and the Brooklyn Tabernacle is prostituted to the infamous uses of a mercenary huckster of a patent nostrum.

Dr. Talmage's sermons are listened to by thousands on Sunday morning, and read on Monday by thousands more. We modestly suggest as a text for some Sunday's discourse: "My house shall be called a house of prayer, but ye have made it a den of thieves."

